# Biallelic *CPAMD8* variants in a patient with ectopia lentis associated with extraocular systemic features reminiscent of Marfan syndrome

**DOI:** 10.1038/s41439-025-00329-9

**Published:** 2025-10-27

**Authors:** Daiju Oba, Mariko Sagara, Sayuri Oda, Miyu Fukushima, Kenta Hasumi, Hirofumi Ohashi

**Affiliations:** Division of Medical Genetics, Saitama Children’s Medical Center, Saitama, Japan

**Keywords:** Disease genetics, DNA sequencing

## Abstract

Here we report an 18-year-old male patient with bilateral ectopia lentis and biallelic *CPAMD8* variants (NM_015692.5:c.[2801delG];[4552C>T]; NP_056507.3:p.[(Gly934GlufsTer64)];[(Gln1518Ter)]). He exhibited previously unreported extraocular features, including a slender build, scoliosis, arachnodactyly and positive thumb sign and wrist sign, which is reminiscent of Marfan syndrome. These findings may suggest that CPAMD8-related disorder is a syndromic condition associated with extraocular systemic features similar to those seen in Marfan syndrome.

*CPAMD8* (MIM 608841) was reported in 2004 as one of the complement component-3 family proteins that are important in innate and acquired immunity^1^. The mRNA of *CPAMD8* gene was shown to be highly expressed in brain, kidney and testis^1^. Thereafter, in 2016, *CPAMD8* abnormality was reported as a cause of autosomal recessive anterior segment dysgenesis, based on observations of four patients from three unrelated families, all of whom exhibited ectopia lentis (EL)^2^. The study also demonstrated that *CPAMD8* mRNA is expressed in the developing embryonic lens, iris, cornea and retina. So far, 16 pathogenic or likely pathogenic variants associated with anterior segment dysgenesis have been registered in ClinVar (https://www.ncbi.nlm.nih.gov/clinvar/), 13 of which are nonsense, frameshift or splice site variants that result in premature termination codons. This suggests that loss of function is the disease-causing mechanism of the gene. Currently, *CPAMD8* is regarded as one of the notable disease-causing genes for EL other than *FBN1*.

To our knowledge, features of *CPAMD8*-related disorder are confined to the eye, and extraophthalmological manifestations have not been reported previously. Here, we report a patient with EL and systemic manifestations reminiscent of Marfan syndrome (MFS) carrying biallelic *CPAMD8* truncating variants.

The patient is a boy who was born to Japanese nonconsanguineous parents after uneventful 31 weeks of gestation. His height, weight and head circumference at birth were 44.5 cm (+1.3 s.d.), 1,940 g (+1.0 s.d.) and 30.3 cm (+0.7 s.d.), respectively. He had no medical concerns including cardiovascular complications before referral. There was no family history of aortic dissection, sudden death or ophthalmological complications. At 15 years of age, he was referred to us by an ophthalmologist because of bilateral EL. His height was 169 cm (+0.1 s.d.), weight 43.7 kg (−1.1 s.d.), body mass index 15.3 kg m^−2^ and arm span 171 cm. He showed features reminiscent of MFS such as a slender build, arachnodactyly and positive thumb sign and wrist sign (Fig. 1A–D). Craniofacial features were also noted, including dolichocephaly, enophthalmos and malar hypoplasia. A spinal radiograph revealed scoliosis (Fig. 1E). The systemic score was calculated as 6 according to the revised Ghent criteria^3^. His parents exhibited no physical features suggestive of MFS. Aortic dilatation, one of the cardinal features of MFS, has not been observed on initial or subsequent annual echocardiograms up to the age of 18 years.

**Fig. 1:**
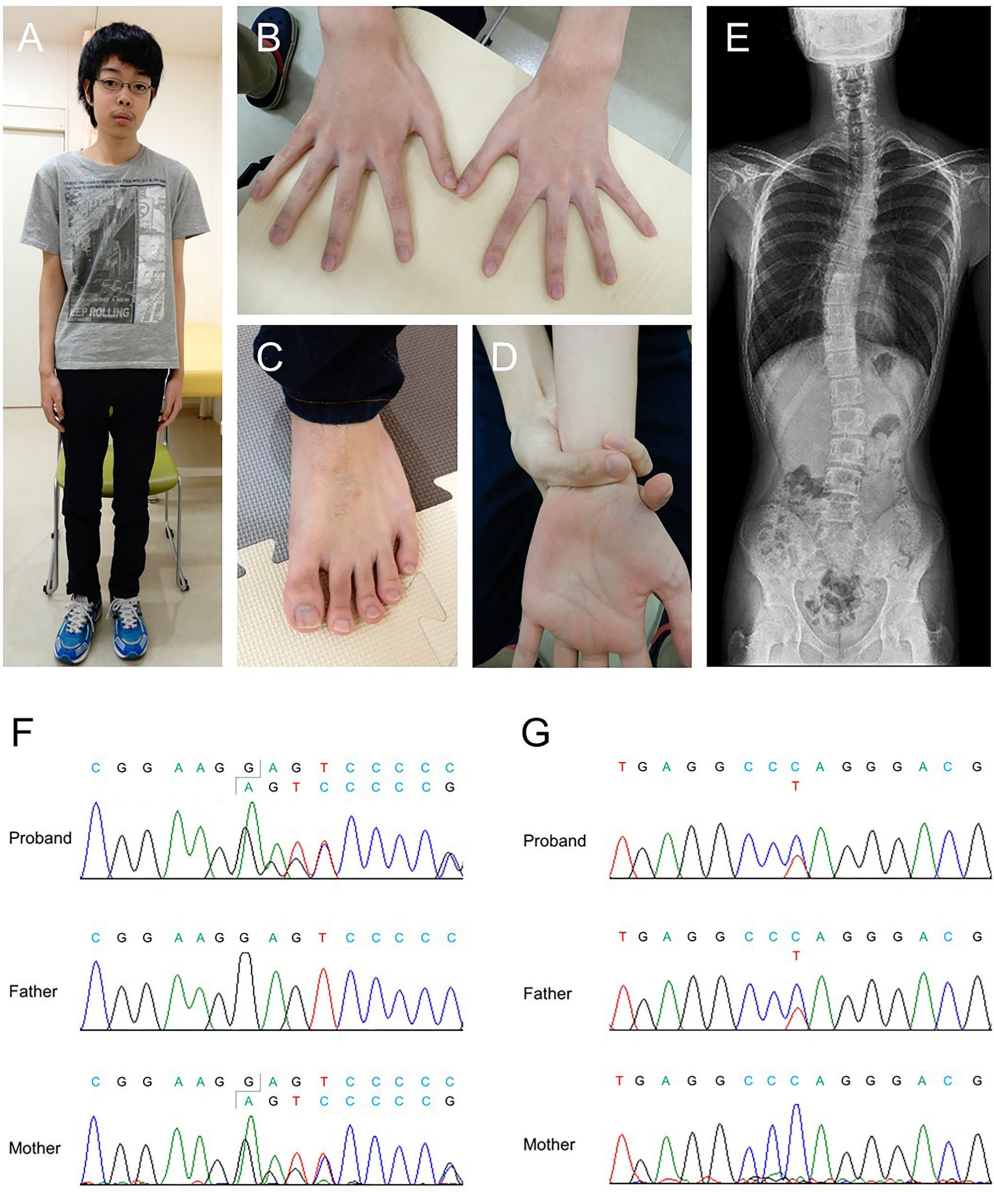
Clinical and molecular findings of the present case. **A**–**E**, Whole body (**A**), hands (**B**), foot (**C**) and positive wrist sign (**D**) photographs and a spine radiograph (**E**) of the proband at the age of 15 years. **F**, **G**, Electropherograms of Sanger sequencing revealing compound heterozygous *CPAMD8* variants of c.2801delG (**F**) and c.4552C>T (**G**).

The targeted sequencing focused on genes associated with MFS and MFS-related disorders revealed no disease-causing variants. Subsequently, we performed whole-exome sequencing and identified three variants in *CPAMD8* (NM_015692.5), c.2801delG;p.(Gly934GlufsTer64), c.3667C>T;p.(Arg1223Cys) and c.4552C>T;p.(Gln1518Ter), in the patient. None of them is found in ToMMo 61KJPN (https://jmorp.megabank.tohoku.ac.jp/), a population database in Japanese. In gnomAD v4.1.0 (https://gnomad.broadinstitute.org/), the allele frequencies of c.2801delG, c.3667C>T and c.4552C>T are 0.0000006339, 0.00001487 and 0.0000006287, respectively. Parental analysis revealed that c.2801delG and c.3667C>T were maternally and c.4552C>T was paternally inherited (Fig. 1F, G). As *CPAMD8*-related disorder is caused by biallelic loss-of-function variants, we concluded that the two biallelic protein-truncating variants, c.2801delG and c.4552C>T, both probably subject to nonsense-mediated mRNA decay, are causative of his *CPAMD8*-related disorder. The c.3667C>T variant, classified as a variant of uncertain significance (PM2_Supporting, PM3_Moderate, PP3_Moderate; REVEL 0.831) per American College of Medical Genetics and Genomics guidelines^4^, is unlikely to be causally related to the patient’s phenotype, as it resides in *cis* with the protein-truncating c.4552C>T variant predicted to undergo nonsense-mediated mRNA decay.

Several cohort studies to explore genetic background of EL using targeted gene panel sequencing have revealed that the vast majority of patients had *FBN1* variants known to cause MFS^5^. Unfortunately, *CPAMD8* was not included in the set of the gene panels used in these studies, probably due to less awareness of the gene as a cause of EL^6,7^ (this also is the case for our laboratory). However, by using more expanded gene panel of 289 genes associated with common heritable anterior eye diseases for sequencing analysis, Chen et al. reported that *CPAMD8* variants were identified in 1.71% of patients in their EL cohort^8^. Thus, when using targeted gene panel sequencing for genetic analysis for patients with EL, *CPAMD8* should be included in the panel.

Our patient had been diagnosed with MFS-related disorder before referral to us, with features reminiscent of MFS. To the best of our knowledge, there have been no previous reports of patients with *CPAMD8*-related disorder presenting with extraocular manifestations, including skeletal features resembling those of MFS, as observed in our patient.

In conclusion, our patient’s findings suggest that *CPAMD8*-related disorder may represent a syndromic condition associated with extraocular systemic features reminiscent of MFS. In patients with EL, whether or not they present with MFS-like features, *CPAMD8* should be included in the genetic analysis. However, it should be noted that this report describes only a single patient. Indeed, we cannot rule out the possibility of a coincidental dual diagnosis, as additional causal variants may remain undetected by the whole-exome sequencing performed in the patient’s analysis. Further accumulation of patients with *CPAMD8*-related disorder, particularly those presenting with extraocular complications, is needed.

## HGV Database

The relevant data from this Data Report are hosted at the Human Genome Variation Database at 10.6084/m9.figshare.hgv.3553. 10.6084/m9.figshare.hgv.3556
